# Additive Manufacturing of Fibre-Reinforced Polymer Composites

**DOI:** 10.3390/polym17121652

**Published:** 2025-06-14

**Authors:** Chengxing Yang, Kui Wang, Jianxun Zhang, Andrea Codolini

**Affiliations:** 1 Key Laboratory of Traffic Safety on Track, Ministry of Education, School of Traffic & Transportation Engineering, Central South University, Changsha 410075, China; kui.wang@csu.edu.cn; 2 State Key Laboratory of Safety and Resilience of Civil Engineering in Mountain Area, East China Jiaotong University, Nanchang 330013, China; 3 National Key Laboratory of Equipment State Sensing and Smart Support, National University of Defense Technology, Changsha 410073, China; 4 State Key Laboratory for Strength and Vibration of Mechanical Structures, School of Aerospace Engineering, Xi’an Jiaotong University, Xi’an 710049, China; jianxunzhang@mail.xjtu.edu.cn; 5 Engineering and Physical Science Research Council, Centre for Innovative Manufacturing in Composites, University of Cambridge, Cambridge CB2 1TN, UK; ac2386@cam.ac.uk

## 1. Introduction

Additive manufacturing (AM) has emerged as a transformative approach to fabricating complex geometries with tailored architectures, offering significant advantages in terms of design freedom, material efficiency, and on-demand production. Among the diverse materials applicable to AM, fibre-reinforced polymer composites (FRPCs) have garnered increasing attention due to their exceptional mechanical properties, high specific strength, and lightweight nature [1,2,3]. The synergistic combination of reinforcing fibres and polymer matrices significantly enhances structural integrity, stiffness, and impact resistance, rendering FRPCs suitable for a wide array of high-performance applications, including aerospace, automotive, marine, biomedical, and consumer products [4,5]. Furthermore, the incorporation of bio-based and biodegradable matrices has elevated FRPCs as promising candidates for sustainable material systems, aligning with global efforts toward carbon neutrality and circular economy strategies [6].

Recent developments in AM technologies have further expanded the potential of FRPCs by enabling the incorporation of both short and continuous fibres within thermoplastic and thermosetting matrices [7,8,9]. Advanced printing techniques such as fused deposition modelling (FDM), stereolithography (SLA), and selective laser sintering (SLS) have been tailored to accommodate fibre reinforcements, offering unprecedented opportunities to control fibre orientation, layer thickness, and part topology [10,11]. Moreover, novel strategies, such as curved layer deposition, voxel-level printing, spatially controlled fibre alignment, and hybrid AM processes, have been proposed to overcome traditional challenges in manufacturing fibre-reinforced components, resulting in enhanced mechanical performance and structural optimization [12,13]. High-performance engineering polymers, including polyether ether ketone (PEEK), polyamide (PA), and polylactic acid (PLA), have been successfully integrated into AM platforms, thereby extending the application scope of FRPCs to demanding environments, such as aerospace propulsion systems and medical implants [14].

Despite the substantial progress made, several challenges remain in fully realizing the potential of AM-FRPCs. Key issues include controlling the interfacial adhesion between the fibre and matrix, mitigating voids and porosity during printing, managing thermal residual stresses, and addressing the anisotropic behaviour caused by layer-by-layer deposition [15,16]. To address these limitations, recent studies have explored computational simulation, machine learning-assisted process optimization, and real-time monitoring systems to guide printing accuracy and material behaviour [17]. Meanwhile, the use of natural fibres, nanocellulose, and lignocellulosic reinforcements offers eco-friendly alternatives that retain competitive mechanical and thermal performance [18,19]. This Special Issue brings together recent innovations in materials design, processing technologies, and characterization techniques to advance the fundamental understanding and practical implementation of additive manufacturing for fibre-reinforced polymer composites.

## 2. Overview of the Published Articles

The Special Issue “Additive Manufacturing of Fibre-Reinforced Polymer Composites” brings together 13 impactful studies that delve into various facets of additive manufacturing (AM) applied to fibre-reinforced polymer composites (FRPCs). These contributions span innovations in mechanical property enhancement, process optimization, material sustainability, and smart sensing functionalities. Each article provides valuable insights that collectively represent the forefront of this rapidly evolving field.

The study by Wang et al. (Contribution 1) focuses on improving the interlaminar fracture toughness of aramid fibre/epoxy resin composites by incorporating laser-induced graphene (LIG) and short Kevlar fibres. A major bottleneck for aramid composites in structural applications is their poor Mode II interfacial toughness. Through Mode II and tensile tests, this research shows that LIG, combined with Kevlar fibres, significantly enhances toughness by 381.60%. More impressively, LIG offers a functional role in damage monitoring due to its piezoresistive properties, enabling the real-time resistance-based detection of delamination and crack propagation. Although LIG slightly reduces the tensile strength, this drawback is mitigated by the Kevlar additions. SEM analysis confirms enhanced surface roughness and fibre bridging, indicating improved crack deflection pathways. This multifunctional strategy represents a notable step toward intelligent FRPCs capable of both load bearing and structural health monitoring.

Zhang et al.’s (Contribution 2) work provides an in-depth evaluation of glass fibre-reinforced polymer (GFRP) main spars for wind turbine blades containing artificial delamination and wrinkle defects. By combining tensile testing with acoustic emission (AE) monitoring, the study analyses how these defects evolve under load and affect mechanical integrity. Using K-means clustering of AE data, the research successfully classifies different damage modes and correlates them with microstructural observations. Notably, the AE signature and characteristic frequencies remain consistent across different defect types, suggesting a degree of universality in GFRP damage responses. The study offers practical guidance for condition-based monitoring in renewable energy structures and contributes significantly to our understanding of defect-driven failure in FRPCs.

Andreozzi et al. (Contribution 3) investigates the buckling behaviour of isogrid structures manufactured using continuous carbon fibre-reinforced polymers via an Anisoprint Composer A3. This paper demonstrates that increasing the infill density enhances the buckling resistance and transitions failure from local to global buckling, leading to more uniform stress distribution. However, SEM and optical analyses highlight voids as a persistent issue that undermines the mechanical performance. By employing coextrusion technology and geometrically optimized structures, this study bridges the gap between high-performance aerospace-grade components and cost-effective 3D printing processes. The findings are especially relevant for aerospace and automotive industries seeking weight-efficient, load-bearing printed components.

Stepanov et al. (Contribution 4) addresses the challenges associated with 3D printing high-viscosity, glass fibre-filled PEEK composites. Using a combination of Taguchi design, FEM simulation, and artificial neural networks, this study identifies the optimal printing parameters (460 °C extruder temp, 20 mm/min travel speed, and 4 rpm screw rotation) that yield homogeneous microstructures and low porosity. Computed tomography validates these parameters, showing porosity levels under 10%. Furthermore, strategies for fibre alignment and post-printing treatment are proposed to enhance mechanical performance. This comprehensive approach to parameter tuning sets a benchmark for high-temperature engineering polymers and their applications in biomedical and aerospace components.

Wang et al. (Contribution 5) contributes an analytical and numerical framework for assessing the low-velocity impact behaviour of clamped rectangular sandwich tubes composed of fibre metal laminates (FMLs) and foam cores. Using a modified rigid plastic model, the study evaluates the effects of the metal volume fraction, fibre content, and foam strength on impact resistance. Validation against finite element simulations confirms the model’s predictive accuracy. The results reveal that a higher fibre content and foam strength substantially enhance energy absorption and the load-bearing capacity. This study not only informs the structural design of impact-resistant components, but also showcases the synergy between fibre layups and hybrid core structures.

Almeida (Contribution 6) explores the development of eco-friendly FFF filaments by reinforcing polylactic acid (PLA) with cocoa husk-derived cellulose fibres. Both untreated (UCFF) and chemically treated (TCFF) fibres are investigated, with TCFF yielding an 18% improvement in tensile strength. Through SEM, TGA, and FTIR analyses, the study reveals that chemical treatment enhances fibre–matrix interactions and thermal stability. ANOVA confirms the statistical significance of these improvements, and the high R2 values underscore the robustness of the experimental design. This work highlights the viability of agricultural waste as a reinforcement for AM biocomposites, offering a sustainable path forward in reducing the reliance on petroleum-based materials.

Zhang et al. (Contribution 7) explored the potential of integrating continuous carbon fibres (CCFs), short carbon fibres (SCFs), and short glass fibres (SGFs) within polyamide (PA) and polylactide (PLA) matrices to design high-performance I-beam structures using AM. Employing a multi-objective optimization approach grounded in the NSGA-II algorithm, the study developed three I-beam types: primitive (P-type), designed (D-type), and optimized (O-type). Mechanical testing demonstrated remarkable improvements in the stiffness-to-mass and load-to-mass ratios, with optimized I-beams exhibiting increases of 30.05% and 40.59%, respectively. The study underscored the effectiveness of structural optimization in enhancing AM-fabricated composite components and emphasized the critical role of fibre type and matrix selection in tailoring mechanical performance.

Wang et al. (Contribution 8) presented a systematic investigation into the flexural behaviour of PLA-based composite panels fabricated via multi-material fused filament fabrication (FFF). Three PLA derivatives, including foam agent-modified and glass fibre-reinforced eco-friendly variants, were combined in varying sequences and ratios. The Taguchi method was used to analyze the influence of four parameters: material sequence, relative volume ratio, filling pattern, and filling density. The study identified the optimal configurations for maximizing the bending strength and modulus of elasticity. The material sequence emerged as the most influential factor, with performance varying up to 60% depending on the configuration. The validated results provide important guidelines for future multi-material 3D printing applications, particularly in lightweight structural design.

Niu et al. (Contribution 9) addressed the challenge of enhancing the toughness and durability of cement-based composites by incorporating multi-walled carbon nanotubes and polypropylene fibres. Through comprehensive mechanical testing and microstructural analysis, the study revealed that MWCNTs improve compressive strength and reduce mass loss, while PP fibres increase flexural performance and resist crack propagation. The synergistic effect of MWCNTs and PP fibres led to improved internal densification and pore refinement. Notably, the optimized composites showed a 19.1% increase in flexural strength and a reduction of more than 25% in key durability indicators. This study provides a practical pathway for developing advanced, fibre-modified, cement-based composites for construction and infrastructure.

Wang et al. (Contribution 10) examined how printing path strategies affect the structural integrity of continuous fibre-reinforced polymer honeycomb structures (CFRPHSs) fabricated using FDM. By experimenting with multiple path configurations, the team found that fibre dislocations at path corners resulted in stiffness variations and localized weaknesses. Among the tested geometries, the staggered trapezoidal path yielded the best mechanical outcomes, achieving the highest specific load capacity (68.33 N/g) and flexural stiffness (627.70 N/mm). The study concluded that printing path planning plays a vital role in ensuring mechanical uniformity and enhancing the load-bearing capacity in CFRPHSs, contributing valuable insights into structural design optimization. Zhang et al. (Contribution 11) proposed a novel approach to fabricating multilayer truss structures using continuous fibre-reinforced thermoplastic composites and spatial 3D printing. The design enabled the creation of pyramid trusses with tunable density and directionally extended layers. Experimental tests showed high specific stiffness (up to 401.91 MPa) and compressive strength (30.26 MPa), with relative densities as low as 1.45%. The flexibility of layer-by-layer design allowed for targeted performance customization based on application demands. This work highlights spatial 3D printing as a promising route for creating multifunctional structures in aerospace, civil engineering, and other sectors requiring low-weight, high-efficiency materials.

Baranowski et al. (Contribution 12) introduced a simulation-driven strategy to optimize a new laser-sintering platform for fabricating continuous carbon fibre-reinforced polymer parts. Using COMSOL Multiphysics, the authors evaluated the heat distribution and fibre–matrix integration efficiency. The optimized process significantly reduced the width and depth of the heat-affected zone (by 56% and 44%, respectively) and shortened the fibre integration time by over 230%. This contribution demonstrates the value of numerical modelling in refining the AM process parameters and enhancing throughput, especially for high-strength, low-batch components in aerospace and defence. Bogusz (Contribution 13) offered a high-resolution, experimental investigation into the in-plane shear behaviour of GFRP composites using ±45° off-axis tension tests and digital image correlation (DIC). The non-contact DIC technique enabled the visualization of strain fields and crack initiation in real time. The study identified microcrack formation and strain localization as contributors to shear nonlinearity. The comparison with strain gauge data validated the methodology and highlighted the importance of sensor placement in mechanical analysis. The findings provide crucial insights into failure mechanisms and serve as a benchmark for validating finite element models of fibre-reinforced composites.

Collectively, these 13 articles exemplify the current trajectory of research in the additive manufacturing of fibre-reinforced polymer composites. From structural optimization and sustainable biocomposites to simulation-enhanced process control and advanced characterization, each study contributes distinctively to the scientific and practical understanding of next-generation AM materials and methods.

## 3. Conclusions and Outlooks

In conclusion, the research contributions featured in this Special Issue collectively underscore the dynamic progression of additive manufacturing (AM) in the realm of fibre-reinforced polymer composites (FRPCs). From advanced structural design and process parameter optimization to the development of multifunctional and sustainable composite systems, these studies demonstrate the diverse capabilities of AM technologies to meet the evolving demands of high-performance applications. Notably, the integration of continuous and short fibres into thermoplastic matrices, the use of eco-friendly reinforcements, and the adoption of simulation-guided approaches have significantly advanced both the mechanical performance and functional sophistication of AM-FRPC components. Moreover, the contributions highlighting the role of artificial intelligence in process optimization and digital image correlation in failure analysis signal a trend toward data-driven, digitally informed manufacturing ecosystems.

Looking forward, the future of AM-FRPCs will likely be shaped by several strategic research directions. Firstly, there is a pressing need to develop scalable AM processes that ensure consistent fibre alignment, void-free structures, and robust interfacial bonding. This can be achieved by integrating real-time monitoring, closed-loop control systems, and in situ process diagnostics. Secondly, the exploration of hybrid material systems, such as bio-based polymers combined with advanced synthetic fibres, holds promise for balancing sustainability and performance. Thirdly, multi-material and functionally graded composites fabricated via voxel-level or multi-axis AM platforms could unlock next-generation components with spatially tuned properties. Additionally, machine learning and physics-informed models can further support intelligent process planning and quality assurance. Finally, standardization in testing protocols, lifecycle assessments, and the long-term performance evaluation of AM-FRPCs will be critical for accelerating their adoption in safety-critical industries such as the aerospace, energy, and biomedical sectors. By addressing these opportunities, the research community can continue to push the boundaries of what is achievable through the additive manufacturing of fibre-reinforced composites.
